# *Naja atra* Cardiotoxin 1 Induces the FasL/Fas Death Pathway in Human Leukemia Cells

**DOI:** 10.3390/cells10082073

**Published:** 2021-08-12

**Authors:** Jing-Ting Chiou, Liang-Jun Wang, Yuan-Chin Lee, Long-Sen Chang

**Affiliations:** 1 Institute of Biomedical Sciences, National Sun Yat-Sen University, Kaohsiung 804, Taiwan; d072050008@student.nsysu.edu.tw (J.-T.C.); d052050011@student.nsysu.edu.tw (L.-J.W.); d042050010@student.nsysu.edu.tw (Y.-C.L.); 2 Department of Biotechnology, Kaohsiung Medical University, Kaohsiung 807, Taiwan

**Keywords:** cardiotoxin, p38 MAPK, c-Jun/ATF-2, FasL, Fas

## Abstract

This study aimed to investigate the mechanistic pathway of *Naja atra* (Taiwan cobra) cardiotoxin 1 (CTX1)–induced death of leukemia cell lines U937 and HL-60. CTX1 increased cytoplasmic Ca^2+^ and reactive oxygen species (ROS) production, leading to the death of U937 cells. It was found that Ca^2+^-induced NOX4 upregulation promoted ROS-mediated p38 MAPK phosphorylation, which consequently induced c-Jun and ATF-2 phosphorylation. Using siRNA knockdown, activated c-Jun and ATF-2 were demonstrated to regulate the expression of Fas and FasL, respectively. Suppression of Ca^2+^-mediated NOX4 expression or ROS-mediated p38 MAPK activation increased the survival of U937 cells exposed to CTX1. FADD depletion abolished CTX1-induced cell death, caspase-8 activation, and t-Bid production, supporting the correlation between the Fas death pathway and CTX1-mediated cytotoxicity. Among the tested *N. atra* CTX isotoxins, only CTX1 induced Fas and FasL expression. Chemical modification studies revealed that intact Met residues were essential for the activity of CTX1 to upregulate Fas and FasL expression. Taken together, the data in this study indicate that CTX1 induces c-Jun-mediated Fas and ATF-2-mediated FasL transcription by the Ca^2+^/NOX4/ROS/p38 MAPK axis, thereby activating the Fas death pathway in U937 cells. Furthermore, CTX1 activates Fas/FasL death signaling in the leukemia cell line HL-60.

## 1. Introduction

The venom of snakes from the family *Elapidae* contains a large amount of cardiotoxins (CTXs) [1]. Although CTXs and α-neurotoxins from elapid snake venom share similar three-loop folded structures, they have different biological activities and amino acid sequence variations [2,3]. Recent studies have revealed that the blockage of negatively charged carboxyl groups in cobrotoxin, a *Naja atra* α-neurotoxin, renders the toxin molecule to demonstrate CTX-like activities [4]. This indicates that amino acid substitutions drive the divergence in the biological activities of CTXs and α-neurotoxins during the evolution of snake venom proteins. Unlike snake α-neurotoxins, which specifically block postsynaptic nicotinic acetylcholine receptors, CTXs exhibit various functions, including myotoxicity, cardiotoxicity, hemolytic activity, tumoricidal activity, and protease activity enhancement [1,5,6,7,8]. Accumulated evidence supports that the cell membrane is the main target of CTXs [9]. However, membrane-disrupting activity cannot fully elucidate the cytotoxicity of CTXs [7]. Several studies have reported that internalized CTXs target mitochondria or lysosomes to display their cytotoxicity [10,11,12,13]. A previous study suggested that the binding of *N. atra* CTX3 with cell surface receptor(s) activates apoptosis signaling in lymphocytes [14]. Notably, these cellular targets cannot fully explain the diversity of the biological activities in CTXs. Therefore, one can determine whether there may be some undetermined targets or pathways involved in the biological activities of CTXs, specifically when different types of cells are targeted by the toxins. Proteomic studies have revealed that the venom of a single *N. atra* snake contains many CTX isotoxins [15]. Chromatographic separation of *N. atra* venom has identified six CTX isotoxins [16]. All *N. atra* CTXs show a high degree of sequence conservation, but they do not have the same cytotoxicity and membrane-damaging activity [17,18,19]. These observations suggest that slight changes in the amino acid residues of CTXs have significant impact on biological activities. It is well documented that CTXs induce apoptotic death in cancer cells, but the detailed mechanism remains to be fully resolved [20,21]. Cell apoptosis is mediated through death receptor-mediated or mitochondria-mediated pathways [22]. TNF-α, FasL, TRAIL, and their receptors are key players in death receptor-mediated apoptosis [23,24]. Interestingly, an in vivo study showed that injecting *N. atra* CTX into the muscles of mice causes increased expression of Fas mRNA in skeletal muscle cells [25]. Therefore, there may be a connection between the cytotoxicity of *N. atra* CTXs and the Fas-mediated apoptosis pathway. Several studies have indicated that leukemia U937 and HL-60 cells tend to undergo FasL/Fas-mediated death pathways when exposed to snake venom phospholipase A_2_ and natural compounds [26,27,28,29]. Therefore, we used U937 and HL-60 cells to explore the effect of *N. atra* CTXs on the expression of Fas and FasL in these cell lines. Interestingly, it was found that *N. atra* CTX1 was the only CTX that induced FasL/Fas-mediated apoptosis pathway. Treatment with CTX1 also caused Fas-mediated apoptosis in leukemia HL-60 cells. Furthermore, we investigated the mechanism by which CTX1 induced the upregulation of Fas and FasL.

## 2. Materials and Methods

### 2.1. Reagents

*N. atra* CTXs were prepared in our laboratory [8]. The Met residues and carboxyl groups of CTX1 were modified with chloramine-T and semicarbazide, respectively [18,19]. The three Met residues at positions 24, 26, and 27 were oxidized by chloramine-T in Met-modified CTX1 [18]. Semicarbazide-modified CTX1 (SEM-CTX1) contains semicarbazide-conjugated Asp29 and Asp40 [19]. Without specific indication, the reagents used in this study were obtained from Sigma-Aldrich (St. Louis, MO, USA), and ML171 and GLX351322 were from MedChem Express (Monmouth Junction, NJ, USA). Z-VAD-FMK, Z-DEVD-FMK, and Z-IETD-FMK were obtained from Calbiochem (San Diego, CA, USA), and H_2_DCFDA, Fluo-4 AM, tetramethylrhodamine methyl ester (TMRM), annexin V-FITC kit, and BAPTA-AM were from Molecular Probes (Carlsbad, CA, USA).

### 2.2. Cell Culture and Cell Viability Assay

Human AML U937 and HL-60 cells from Bioresource Collection and Research Center (Hsinchu, Taiwan) were cultured in RPMI 1640 medium containing 10% FCS, 1% sodium pyruvate, 2 mM glutamine, and 1% penicillin/streptomycin. The cells were maintained at 37 °C in humidified air containing 5% CO_2_. Cell culture supplements were purchased from GIBCO/Life Technologies Inc. (Carlsbad, CA, USA). The survival rate of the cells was determined using an MTT assay. Exponentially growing cells (5 × 10^4^) were plated in each well of a 96-well plate and treated with 0 to 1000 nM CTXs in serum-free medium for 4 h. MTT solution (1 mg/mL) was then added to each well and incubated for 4 h. After removing the supernatant from the wells, the formazan crystals produced by the reduction of MTT were dissolved by addition of 100 μL DMSO per well. The absorbance was measured at 595 nm using a plate reader.

### 2.3. Apoptosis Assay

CTX1-treated cells were stained with annexin V-FITC kit (containing annexin V and propidium iodide (PI); Molecular Probes) according to the manufacturer’s instructions. U937 cells (1 × 10^6^ per well) plated in 6-well plates were treated with 500 nM CTX1 for 4 h. CTX1-treated cells were washed with cold PBS and resuspended with binding buffer (10 mM Hepes, pH 7.4, 140 mM NaCl, 2.5 mM CaCl_2_). Then 5 μL of annexin V-FITC and 5 μL of PI were added, and the cells were incubated for 15 min in the dark. Apoptotic cell death was detected using flow cytometer. On flow cytometric scatter graphs, the left lower quadrant represents remaining live cells. The right lower quadrant represents the population of early apoptotic cells. The right upper quadrant represents the accumulation of late apoptotic cells.

### 2.4. Intracellular Ca^2+^ Measurement

Cells (6 × 10^5^ in each well of a 12-well plate) were treated with 500 nM CTX1 (for U937 cells) or 800 nM (for HL-60 cells) for indicated time periods. CTX1-treated cells were loaded with 5 μM Fluo-4 AM for 1 h. The fluorescent signal of intracellular Ca^2+^ was detected using a fluorescence plate reader. The data were expressed as a fold change compared to the fluorescence intensity of untreated cells.

### 2.5. Detection of Cellular ROS and Mitochondrial Depolarization

Cells (6 × 10^5^ in each well of a 12-well plate) were treated with 500 nM CTX1 (for U937 cells) or 800 nM (for HL-60 cells) for indicated time periods. CTX1-treated cells were loaded with 10 μM H_2_DCFDA or 2 nM TMRM at 25 °C for 20 min. The fluorescence signal intensity of H_2_DCFDA measured with a fluorescence plate reader was used to evaluate the relative ROS levels. TMRM fluorescence signal was analyzed using a flow cytometer. Decreased TMRM fluorescence indicates dissipation of the mitochondrial membrane potential (ΔΨm).

### 2.6. Western Blot Analysis

The cells (1 × 10^6^ in each well of a 6-well plate) were treated with 500 nM (for U937 cells) or 800 nM (for HL-60 cells) CTX1 for 4 h. CTX1-treated cells were lysed in RIPA buffer containing protease and phosphatase inhibitors. The Bradford method was used to measure protein concentration. An equal amount of protein (15 μg) was separated by SDS-PAGE and transferred to PVDF membranes by the immunoblotting, as described in previous studies [7]. The membranes were blocked with non-fat milk, followed by incubation with primary antibodies and HRP-labeled secondary antibodies. The antibodies recognized caspase-3, caspase-8 (Calbiochem, San Diego, CA, USA), MCL1, p-c-Fos (Ser374), Fas, FADD (Santa Cruz Biotechnology, Santa Cruz, CA, USA), FasL, p-c-Jun (Ser73), c-Jun, p-ATF-2 (Thr71), ATF-2, c-Fos, p-ERK, ERK, p-p38 MAPK, p38 MAPK, p-JNK, JNK, BCL2, BAX (Cell Signaling Technology, Beverly, MA, USA), BCL2L1, BAK, BID, PARP (BD Pharmingen, San Jose, CA, USA), and β-actin antibody (Sigma-Aldrich), which were used in this study. HRP-labeled secondary antibodies were the products of Sigma-Aldrich. Immunoreactive bands were visualized using chemiluminescent substrates (Perkin Elmer, Waltham, MA, USA). The experiments were performed at least in triplicates, and representative blots are shown. The results of western blots were quantified by a scanning densitometer. The β-actin is used as a loading control, and quantitative analyses of the protein levels are indicated at the immunoblots.

### 2.7. Quantitative RT-PCR (qRT-PCR)

The cells (1 × 10^6^ in each well of a 6-well plate) were treated with 500 nM (for U937 cells) or 800 nM (for HL-60 cells) CTX1 for 4 h. Total RNA was extracted using the RNeasy Mini Kit (QIAGEN, Leiden, The Netherlands), and reverse-transcribed to cDNA using M-MLV reverse transcriptase (Promega, Madison, WI, USA). PCR amplification of cDNA was carried out using the GoTaq qPCR Master mix (Promega). The relative changes in gene expression were evaluated using the 2^−^^ΔΔCT^ method. The GAPDH gene transcript was used as an internal control for the normalization of gene expression. The primers used are listed in Supplementary Table S1.

### 2.8. Promoter Activity Assay

The luciferase constructs, pGVB-Fas and HsLuc-FasL pFLF1, were described in previous studies [28]. The pGL3-NOX4 plasmid was a generous gift from Dr. V.J. Thannickal (University of Alabama, USA). The luciferase constructs were transfected into cells (1 × 10^6^ in each well of a 6-well plate) using TurboFect^™^ Transfection Reagent (ThermoFisher Scientific, Waltham, MA, USA). At 24 h post-transfection, the cells were exposed to 500 nM (for U937 cells) or 800 nM (for HL-60 cells) CTX1 for an additional 4 h. Afterwards, cells were harvested for luciferase activity assay. Luciferase activity was measured using a dual-luciferase reporter assay system kit (Promega) and normalized to *Renilla* luciferase activity.

### 2.9. Transient Transfection of siRNA

The synthesized c-Jun, ATF-2, FADD, and negative control siRNAs were obtained from Santa Cruz Biotechnology, and siRNAs were transfected into cells (1 × 10^6^ in each well of a 6-well plate) using TurboFect^™^ Transfection Reagent. At 24 h post-transfection, the cells were exposed to 500 nM (for U937 cells) or 800 nM (for HL-60 cells) CTX1 for an additional 4 h. Afterwards, cells were harvested for western blot analysis or luciferase activity assay.

### 2.10. Statistical Analysis

Results are presented as the mean ± SD and all data were repeated in triplicates. Data were analyzed using Student’s *t*-test, and differences with *p* <  0.05 were considered statistically significant

## 3. Results

### 3.1. CTX1 Induces Fas and FasL Upregulation in U937 Cells

**Figure 1:**
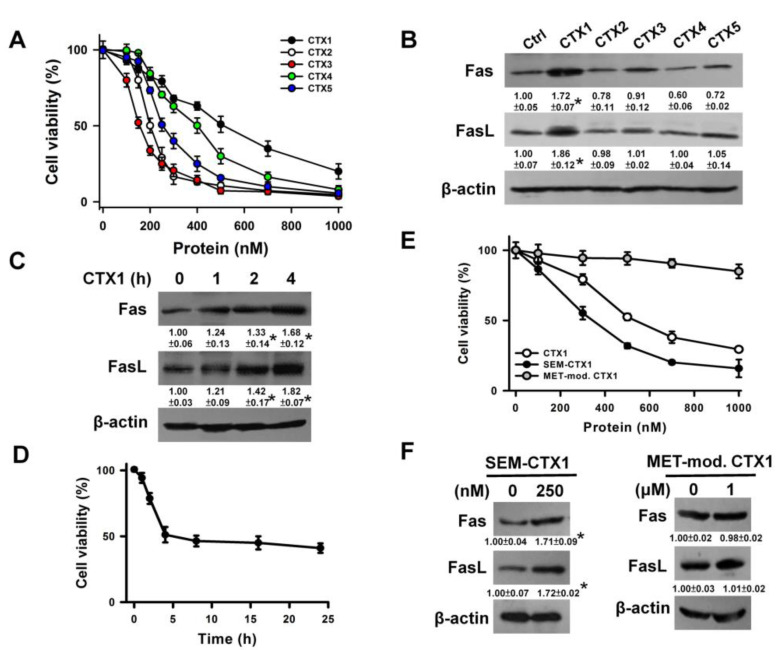
Upregulation of Fas and FasL in CTX1-treated U937 cells. (**A**) CTX1, CTX2, CTX3, CTX4, and CTX5 induced cell death in a concentration-dependent manner. U937 cells were incubated with varying concentrations of CTXs for 4 h. Cell viability was determined by MTT assay. Results are expressed as the percentage of cell proliferation relative to the control. Each value is the mean ± SD of triplicate determinations. (**B**) Effect of CTXs on Fas and FasL protein expression. U937 cells were treated with 500 nM CTX1, 200 nM CTX2, 150 nM CTX3, 400 nM CTX4, and 250 nM CTX5 for 4 h (* *p* < 0.05, CTX1-treated cells compared to untreated control cells). (**C**) CTX1 treatment increased Fas and FasL protein expression in a time-dependent manner. U937 cells were treated with 500 nM CTX1 for indicated time periods (* *p* < 0.05, CTX1-treated cells compared to untreated control cells). (**D**) CTX1 induced death of U937 cells in a time-dependent manner. U937 cells were treated with 500 nM CTX1 for indicated time periods. (**E**) Effect of SEM-CTX1 and Met-modified CTX1 on the viability of U937 cells. U937 cells were incubated with varying concentrations of SEM-CTX1 and Met-modified CTX1 for 4 h. (**F**) Effect of SEM-CTX1 and Met-modified CTX1 on Fas and FasL expression. U937 cells were incubated with indicated SEM-CTX1 and Met-modified CTX1 concentration for 4 h (* *p* < 0.05, SEM-CTX1-treated cells compared to untreated control cells).

Treatment of U937 cells with CTXs ranging from 0 to 1000 nM for 4 h showed that the half maximal inhibitory concentrations (IC_50_) of CTX1, CTX2, CTX3, CTX4, and CTX5 were 500, 200, 150, 400, and 250 nM, respectively (Figure 1A). The IC_50_ dose of CTXs was used to examine their effects on Fas and FasL expression in U937 cells. Among the tested CTXs, only CTX1 induced Fas/FasL expression (Figure 1B). Treatment with 500 nM CTX1 time-dependently increased Fas and FasL expression (Figure 1C). CTX1 reduced the viability of U937 cells in a time-dependent manner and caused the maximal decrease in cell survival after 4 h of treatment (Figure 1D). Previous studies have shown that modification of Asp29 and Asp40 with semicarbzide increased the membrane-damaging activity of CTX1 [19], while modification of Met24, 26, and 27 reduced the membrane-damaging activity of CTX1 [18]. We further examined the effect of semicarbazide-modified CTX1 (SEM-CTX1) and Met-modified CTX1 on Fas and FasL expression in U937 cells. Compared with CTX1, SEM-CTX1 showed increased cytotoxicity, whereas modification of Met residues caused a marked drop in cytotoxicity (Figure 1E). SEM-CTX1 induced the upregulation of Fas and FasL, whereas Met-modified CTX1 was unable to increase Fas and FasL expression (Figure 1F).

### 3.2. CTX1 Induces Death Receptor-Mediated Apoptosis in U937 Cells

**Figure 2:**
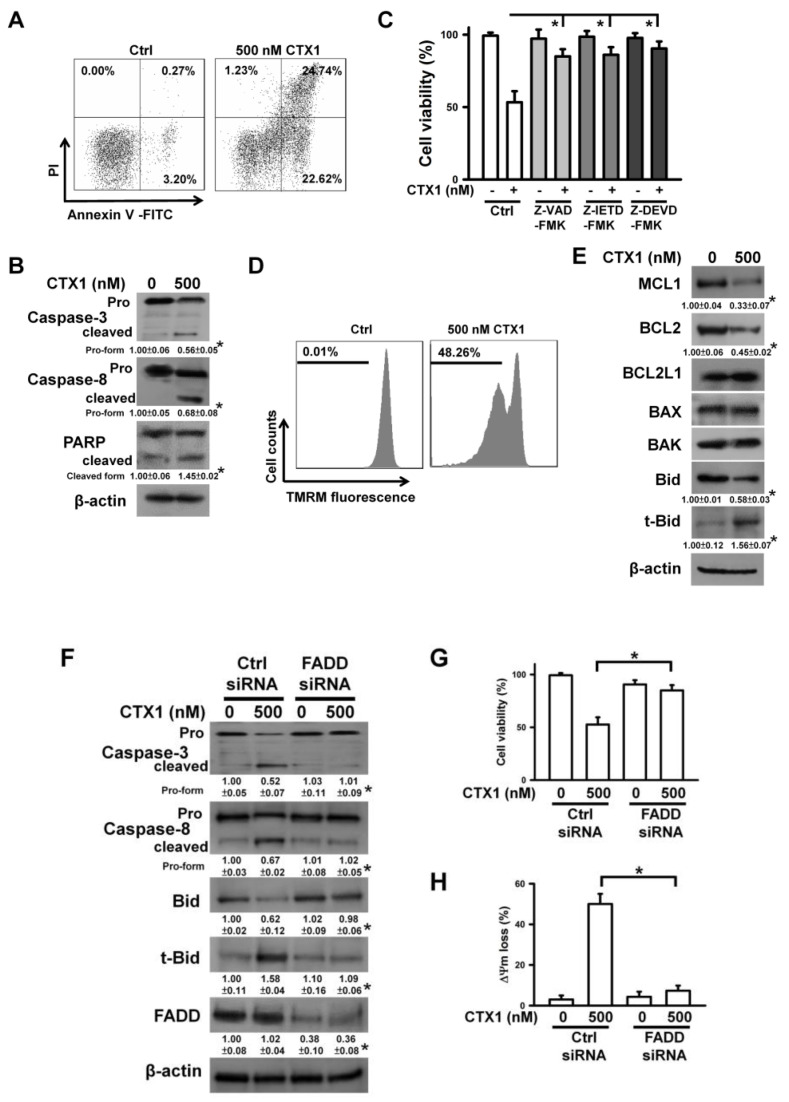
CTX1 induced apoptosis of U937 cells. Without specific indication, U937 cells were treated with 500 nM CTX1 for 4 h. (**A**) Flow cytometry analyses of CTX1-treated U937 cells using annexin V-FITC/propidium iodide double staining. (Left) Untreated control cells. (Right) CTX1-treated U937 cells. (**B**) Western blot analyses showing degradation of procaspases and PARP in CTX1-treated cells (* *p* < 0.05, CTX1-treated cells compared to untreated control cells). (**C**) Viability of CTX1-treated cells was rescued by pretreatment with caspase inhibitors. U937 cells were pretreated with 10 μM Z-VAD-FMK (pan-caspase inhibitor), Z-DEVD-FMK (caspase-3 inhibitor), or Z-IETD-FMK (caspase-8 inhibitor) for 1 h, and then incubated with 500 nM CTX1 for 4 h. Cell viability was determined by MTT. The values represent averages of three independent experiments with triplicate measurement (mean ± SD, * *p* < 0.05). (**D**) Dissipation of mitochondrial membrane potential (ΔΨm) in CTX1-treated cells. The loss of ΔΨm was analyzed by flow cytometry. (**E**) Western blot analyses showing the production of t-Bid and the expression of BCL2 family proteins in CTX1-treated cells (* *p* < 0.05, CTX1-treated cells compared to untreated control cells). (**F**) Transfection of FADD siRNA abrogated CTX1-induced degradation of procaspase-8/-3 and production of t-Bid. U937 cells were transfected with 100 nM control siRNA or FADD siRNA, respectively. After 24 h post-transfection, the cells were treated with 500 nM CTX1 for 4 h (* *p* < 0.05, CTX1-treated FADD siRNA-transfected cells compared to CTX1-treated control siRNA-transfected cells). (**G**) FADD depletion rescued the viability of CTX1-treated cells (mean ± SD, * *p* < 0.05). (**H**) FADD depletion attenuated CTX1-induced ΔΨm loss (mean ± SD, * *p* < 0.05).

Flow cytometry analyses of annexin V- and PI-stained cells showed that CTX1 induced apoptosis in U937 cells (Figure 2A). The cleavage of procaspases-3/-8 and PARP was profoundly increased by CTX1 treatment (Figure 2B). Consistently, caspase inhibitors prevented U937 cells from CTX1-induced death (Figure 2C). This indicates that U937 cells undergo apoptosis after treatment with CTX1. Since FasL/Fas-induced apoptosis resulted in mitochondrial depolarization and caspase-8-mediated t-Bid production [24,30], the effects of CTX1 on ΔΨm and t-Bid production were examined. Treatment of U937 cells with CTX1 induced ΔΨm loss (Figure 2D) and the production of t-Bid (Figure 2E). Moreover, CTX1-treated cells showed reduced expression of MCL1 and BCL2 proteins. It is well documented that FADD recruits procaspase-8 to members of the TNF family death receptors, thus leading to caspase-8 activation that subsequently induces caspase-3-mediated cell apoptosis [24]. Depletion of FADD using siRNA eliminated CTX1-induced production of cleaved caspases-8/-3 and t-Bid (Figure 2F). FADD depletion consistently increased the survival rate of CTX1-treated cells (Figure 2G) and attenuated CTX1-induced ∆Ψm loss (Figure 2H). These results confirmed the correlation between death receptor–mediated apoptosis and CTX1 cytotoxicity.

### 3.3. Ca^2+^-Mediated NOX4 Expression Leads to ROS Generation in CTX1-Treated U937 Cells

**Figure 3:**
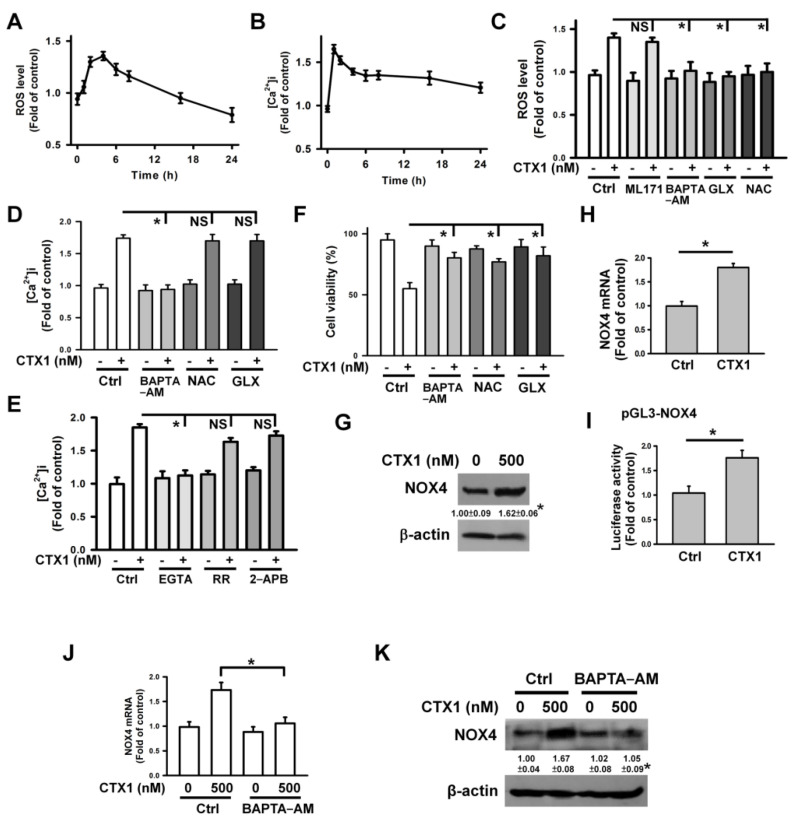
Effect of CTX1 on [Ca^2+^]i and ROS level in U937 cells. U937 cells were treated with 500 nM CTX1 for 2 h (for measuring [Ca^2+^]i) or 4 h (for measuring ROS level and cell viability). On the other hand, U937 cells were pretreated with 2 mM N-acetylcysteine (NAC), 10 μM BAPTA-AM, 10 μM GLX351322, 10 μM ML171, 10 μM EGTA, 10 μM ruthenium red (RR), or 50 μM 2-aminoethoxydiphenyl borane (2-APB) for 1 h and then incubated with 500 nM CTX1 for 2 h (for measuring [Ca^2+^]i) or for 4 h (for measuring ROS level and cell viability). (**A**) CTX1 induced ROS generation in U937 cells. U937 cells were treated with 500 nM CTX1 for indicated time periods. Results were shown as fold-increase in fluorescence intensity compared with the control group. Each value is the mean ± SD of three independent experiments with triplicate measurements. (**B**) CTX1 induced an elevation of [Ca^2+^]i in U937 cells. U937 cells were treated with 500 nM CTX1 for indicated time periods. Results were shown as fold-increase in fluorescence intensity compared with the control group. (**C**) Effect of BAPTA−AM, NAC, ML171, and GLX351322 on CTX1-induced ROS generation (mean ± SD, * *p* < 0.05; NS, not statistically significant). (**D**) Effect of BAPTA−AM, NAC, and GLX351322 on CTX1-induced an increase in [Ca^2+^]i (mean ± SD, * *p* < 0.05; NS, not statistically significant). (**E**) Effect of EGTA, ruthenium red, and 2−APB on CTX1-induced an increase in [Ca^2+^]i (mean ± SD, * *p* < 0.05; NS, not statistically significant). (**F**) Effect of BAPTA−AM, GLX351322, and NAC on the viability of CTX1-treated cells (mean ± SD, * *p* < 0.05). (**G**) Effect of CTX1 on NOX4 protein expression (* *p* < 0.05, CTX1-treated cells compared to untreated control cells). (**H**) Detecting the expression of NOX4 mRNA using qRT-PCR (mean ± SD, * *p* < 0.05). (**I**) Effect of CTX1 on the luciferase activity of NOX4 promoter construct. After transfection with indicated plasmid for 24 h, transfected cells were treated with 500 nM CTX1 for 4 h and then harvested for measuring luciferase activity (mean ± SD, * *p* < 0.05). (**J**) Effect of BAPTA−AM on the expression of NOX4 mRNA in CTX1-treated cells (mean ± SD, * *p* < 0.05). (**K**) Effect of BAPTA−AM on the expression of NOX4 protein in CTX1-treated cells (* *p* < 0.05, BAPTA−AM/CTX1-treated cells compared to CTX1-treated cells).

Since previous studies have indicated that Ca^2+^ and ROS are related to the cytotoxicity of CTXs [7,17], we next analyzed the contribution of intracellular Ca^2+^ concentration ([Ca^2+^]i) and ROS to CTX1 cytotoxicity. CTX1-induced ROS generation reached a maximum level after 4 h of treatment (Figure 3A), while treatment with CTX1 for 2 h increased [Ca^2+^]i to a maximum level in U937 cells (Figure 3B). Chelating intracellular Ca^2+^ with BAPTA-AM eliminated ROS production induced by CTX1 (Figure 3C), while scavenging ROS with NAC did not significantly affect the CTX1-induced increase in [Ca^2+^]i (Figure 3D). These findings indicate that Ca^2+^ induced ROS production in CTX1-treated U937 cells. Compelling evidence has shown that mitochondria and NADPH oxidase (NOX) are the main sources of intracellular ROS [31,32]. Next, we analyzed the effect of NOX inhibitors on the ROS levels. In contrast to ML171 (a NOX1 inhibitor), pretreatment with GLX351322 (a NOX4 inhibitor) inhibited CTX1-induced ROS generation (Figure 3C). GLX351322 did not affect the CTX1-induced increase in [Ca^2+^]i (Figure 3D). Compared to 2-APB and ruthenium red (which inhibited the release of Ca^2+^ from intracellular depots), EGTA markedly inhibited CTX1-induced elevation of [Ca^2+^]i (Figure 3E), suggesting that CTX1 induces the influx of Ca^2+^. Pretreatment with BAPTA-AM, NAC, or GLX351322 inhibited CTX1-induced cell death (Figure 3F). These results corroborate that Ca^2+^-induced ROS production is associated with the cytotoxicity of CTX1 in U937 cells. Since the above data showed NOX4-mediated ROS production in CTX1-treated cells, we further analyzed NOX4 expression in these cells. CTX1 treatment increased NOX4 protein and mRNA expression (Figure 3G,H). Consistent with this, CTX1 treatment increased NOX4 promoter luciferase activity (Figure 3I). Pretreatment with BAPTA-AM abolished CTX1-induced upregulation of NOX4 protein (Figure 3J,K). Altogether, it appeared that Ca^2+^-induced NOX4 expression increased ROS production in CTX1-treated cells.

### 3.4. ROS Induces p38 MAPK Activation in CTX1-Treated U937 Cells

**Figure 4:**
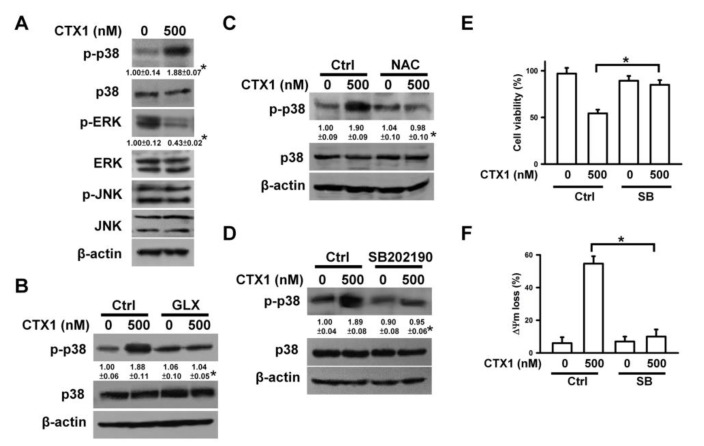
ROS-mediated p38 MAPK activation was associated with CTX1-induced death in U937 cells. U937 cells were directly treated with 500 nM CTX1 for 4 h, or pretreated with 10 μM GLX351322, 2 mM NAC, or 10 μM SB202190 for 1 h and then incubated with 500 nM CTX1 for 4 h. (**A**) Western blot analyses of phosphorylated MAPKs in CTX1-treated U937 cells (* *p* < 0.05, CTX1-treated cells compared to untreated control cells). (**B**) Effect of GLX351322 on CTX1-induced p38 MAPK phosphorylation (* *p* < 0.05, GLX351322/CTX1-treated cells compared to CTX1-treated cells). (**C**) Effect of NAC on CTX1-induced p38 MAPK phosphorylation (* *p* < 0.05, NAC/CTX1-treated cells compared to CTX1-treated cells). (**D**) Effect of SB202190 on CTX1-induced p38 MAPK phosphorylation (* *p* < 0.05, SB202190/CTX1-treated cells compared to CTX1-treated cells). (**E**) Effect of SB202190 on CTX1-induced death of U937 cells (mean ± SD, * *p* < 0.05). (**F**) Effect of SB202190 on CTX1-induced ΔΨm loss in U937 cells (mean ± SD, * *p* < 0.05).

Emerging evidence indicates that ROS can elicit changes in MAPK phosphorylation [33]. Yang et al. [34] reported that MAPK regulates CTX-induced apoptosis. Therefore, the effects of CTX1 on MAPK phosphorylation were analyzed. CTX1 treatment increased p38 MAPK phosphorylation, reduced p-ERK levels, and did not alter p-JNK levels (Figure 4A). CTX1 failed to induce p38 MAPK phosphorylation in U937 cells pretreated with GLX351322, NAC, or SB202190 (a p38 MAPK inhibitor) (Figure 4B–D). SB202190 mitigated CTX1-induced death and ΔΨm loss (Figure 4E,F). These results indicate that ROS-mediated p38 MAPK activation contributes to CTX1-mediated cytotoxicity.

### 3.5. CTX1 Induces p38 MAPK/c-Jun-Mediated Fas Expression and p38 MAPK/ATF-2-Mediated FasL Expression in U937 Cells

**Figure 5:**
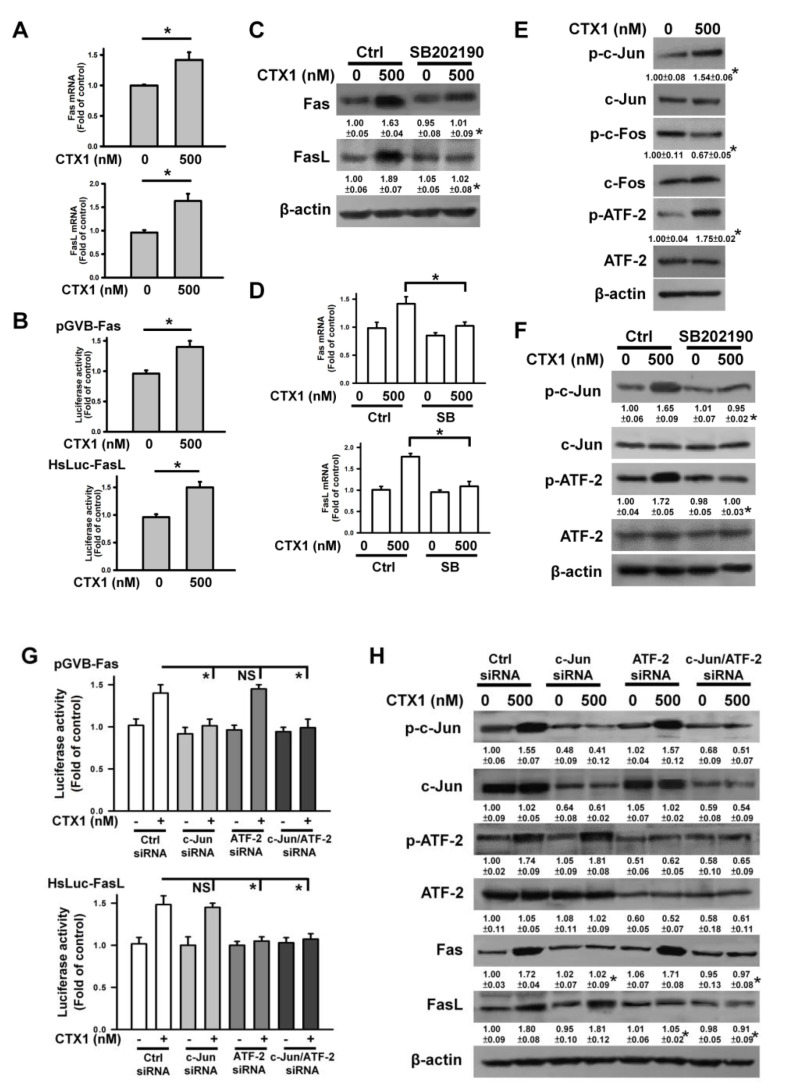
CTX1 induced upregulation of Fas and FasL in U937 cells through p38 MAPK-mediated phosphorylation of c-Jun and ATF-2. U937 cells were directly treated with 500 nM CTX1 for 4 h, or pretreated with 10 μM SB202190 for 1 h and then incubated with 500 nM CTX1 for 4 h. (**A**) Detecting the expression of Fas and FasL using qRT-PCR. qRT-PCR was conducted according to the procedure described in the Materials and Methods section (mean ± SD, * *p* < 0.05). (**B**) CTX1 treatment elicited increase in transcriptional activity of Fas promoter and FasL promoter (mean ± SD, * *p* < 0.05). After transfection with indicated promoter constructs for 24 h, the transfected cells were treated with 500 nM CTX1 for 4 h and then harvested for measuring luciferase activity (mean ± SD, * *p* < 0.05). (**C**) Effect of SB202190 on the expression of Fas and FasL proteins in CTX1-treated cells (* *p* < 0.05, SB202190/CTX1-treated cells compared to CTX1-treated cells). (**D**) Effect of SB202190 on the expression of Fas and FasL mRNAs in CTX1-treated cells (mean ± SD, * *p* < 0.05). (**E**) Effect of CTX1 treatment on levels of p-c-Jun, p-c-Fos and p-ATF-2 (* *p*<0.05, CTX1-treated cells compared to untreated control cells). (**F**) Effect of SB202190 on CTX1-induced c-Jun and ATF-2 phosphorylation (* *p* < 0.05, SB202190/CTX1-treated cells compared to CTX1-treated cells). (**G**) Depletion of c-Jun or ATF-2 using siRNA inhibited the transcriptional activity of Fas and FasL promoter constructs in CTX1-treated U937 cells. U937 cells were co-transfected with Fas promoter/FasL promoter constructs and indicated siRNAs. The used concentration of control siRNA, c-Jun, and ATF-2 was 100 nM. After 24 h post-transfection, the cells were treated with 500 nM CTX1 for 4 h (mean ± SD, * *p* < 0.05; NS, not statistically significant). (**H**) Suppression of c-Jun or ATF-2 expression abrogated CTX1-induced Fas and FasL upregulation in U937 cells. U937 cells were transfected with 100 nM control siRNA, c-Jun siRNA or/and ATF-2 siRNA, respectively. After 24 h post-transfection, the cells were treated with 500 nM CTX1 for 4 h (* *p* < 0.05, CTX1-treated c-Jun siRNA-, ATF-2 siRNA-, or c-Jun siRNA/ATF-2 siRNA-transfected cells compared to CTX1-treated control siRNA-transfected cells).

To examine the mechanism of CTX1-induced upregulation of Fas and FasL, we analyzed their transcription in CTX1-treated cells. CTX1 treatment increased the transcription of Fas and FasL, as demonstrated by qRT-PCR (Figure 5A). Consistently, CTX1 increased Fas and FasL promoter luciferase activity (Figure 5B), suggesting that CTX1 transcriptionally induced Fas and FasL expression. SB202190 pretreatment abolished CTX1-induced increase in Fas/FasL expression (Figure 5C,D), indicating the causal role of p38 MAPK in the CTX1-induced effect. Experimental evidence has demonstrated that phosphorylated c-Jun, c-Fos, and ATF-2 transcriptionally regulate Fas and FasL expression [28,35]. Therefore, we analyzed whether CTX1 affected the phosphorylation of these transcription factors. CTX1 treatment increased p-c-Jun and p-ATF-2 levels, and decreased p-c-Fos levels in U937 cells (Figure 5E). Pretreatment of SB202190 mitigated CTX1-induced phosphorylation of c-Jun/ATF-2 (Figure 5F). Depletion of c-Jun and ATF-2 reduced the ability of CTX1 to increase Fas promoter and FasL promoter luciferase activity, respectively (Figure 5G). Compared with transfection of single siRNA alone, co-transfection of c-Jun and ATF-2 siRNAs reduced Fas and FasL promoter activity to a similar extent. Consistently, downregulation of c-Jun and ATF-2 diminished CTX1-induced expression of Fas and FasL, respectively (Figure 5H). Co-transfection of c-Jun did not further increase the effect of ATF-2 siRNA on suppressing CTX1-induced FasL expression. Similarly, co-transfection with ATF-2 siRNA did not enhance the effect of c-Jun depletion on the inhibition of CTX1-induced Fas expression. Altogether, we concluded that CTX1 elicited p38 MAPK/c-Jun-mediated Fas expression and p38 MAPK/ATF-2-mediated FasL expression in U937 cells.

### 3.6. CTX1 Induces Ca^2+^/NOX4/ROS axis-Mediated p38 MAPK Activation in HL-60 Cells

**Figure 6:**
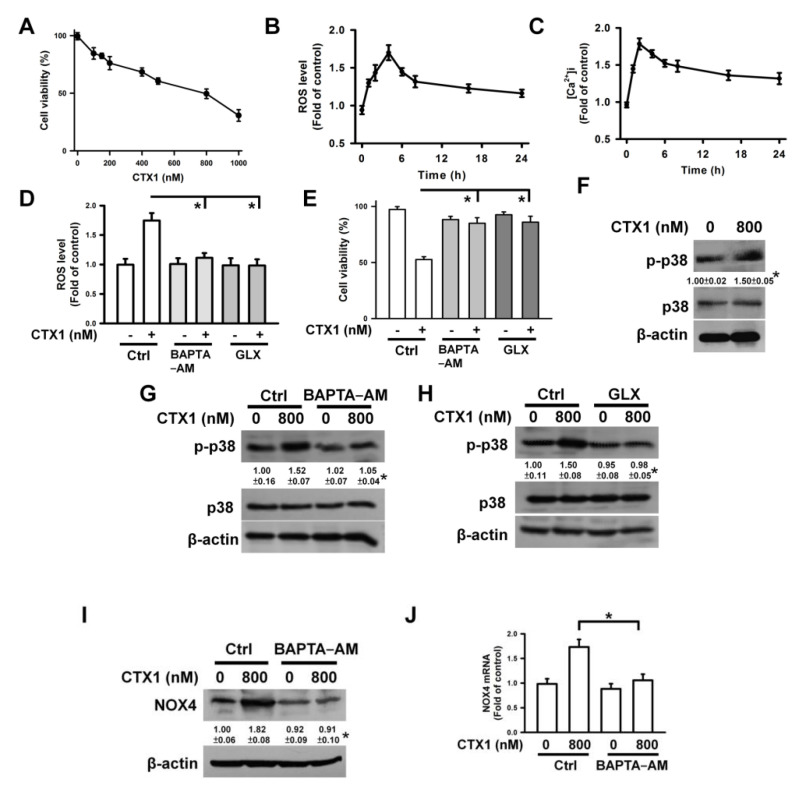
CTX1 induced p38 MAPK phosphorylation in HL-60 cells via the Ca^2+^/NOX4/ROS axis. Without specific indication, HL-60 cells were treated with 800 nM CTX1 for 3 h (for measuring [Ca^2+^]i) or 4 h (for measuring ROS level, cell viability, NOX4 expression, and p-p38 MAPK level). On the other hand, HL-60 cells were pretreated with 10 μM BAPTA-AM or 10 μM GLX351322 for 1 h and then incubated with 800 nM CTX1 for 3 h (for measuring [Ca^2+^]i) or for 4 h (for measuring ROS level, cell viability, NOX4 expression, and p-p38 MAPK level). (**A**) CTX1 induced HL-60 cell death in a concentration-dependent manner. CTX1 induced (**B**) ROS generation and (**C**) an elevation of [Ca^2+^]i in HL-60 cells. HL-60 cells were treated with 800 nM CTX1 for indicated time periods. (**D**) Effect of BAPTA−AM and GLX351322 on CTX1-induced ROS generation (mean ± SD, * *p* < 0.05). (**E**) Effect of BAPTA−AM and GLX351322 on the viability of CTX1-treated HL-60 cells (mean ± SD, * *p* < 0.05). (**F**) CTX1 induced phosphorylation of p38 MAPK in HL-60 cells (* *p* < 0.05, CTX1-treated cells compared to untreated control cells). Effect of (**G**) BAPTA−AM and (**H**) GLX351322 on CTX1-induced p38 MAPK phosphorylation (* *p* < 0.05, BAPTA−AM/CTX1-treated cells compared to CTX1-treated cells; * *p* < 0.05, GLX351322/CTX1-treated cells compared to CTX1-treated cells). Effect of BAPTA−AM on NOX4 protein (**I**) and mRNA (**J**) expression in CTX1-treated cells (* *p* < 0.05, BAPTA−AM/CTX1-treated cells compared to CTX1-treated cells).

To examine whether CTX1 also increased Fas/FasL expression in other cell lines, we investigated its effect on HL-60 cells. CTX1 reduced the viability of HL-60 cells by 50% at a concentration of approximately 800 nM after 4 h of treatment (Figure 6A). A single dose was used to analyze the cytotoxicity of CTX1 in HL-60 cells. CTX1 induced a maximal increase in ROS generation after 4 h treatment (Figure 6B), while a maximal increase in [Ca^2+^]i was observed after 3 h of CTX1 treatment (Figure 6C). Pretreatment with BAPTA-AM or GLX351322 eliminated ROS generation and cell death induced by CTX1 (Figure 6D,E), suggesting the involvement of Ca^2+^ and ROS in CTX1-induced HL-60 cell death. CTX1 induced p38 MAPK activation (Figure 6F), while BAPTA-AM or GLX351322 blunted this CTX1-induced effect (Figure 6G,H). Moreover, BAPTA-AM abolished the CTX1-induced increase in NOX4 protein and mRNA expression (Figure 6I,J). These results indicate that CTX1 induces Ca^2+^/NOX4/ROS axis-mediated p38 MAPK activation in HL-60 cells.

### 3.7. CTX1 Induces the FasL/Fas Death Pathway in HL-60 Cells

**Figure 7:**
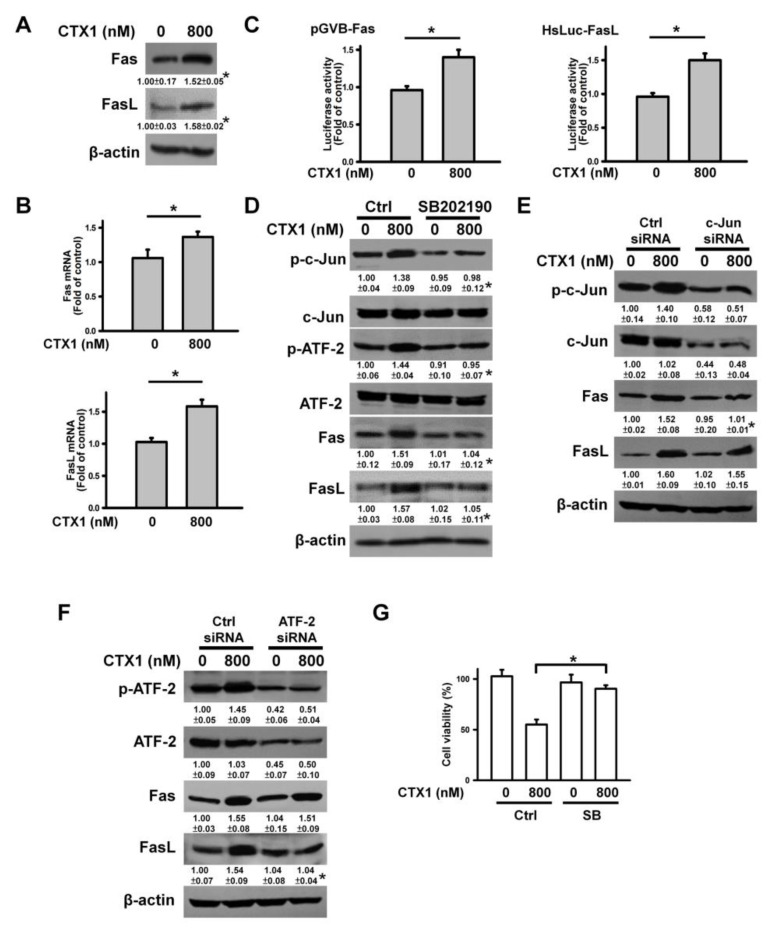
CTX1 induced the upregulation Fas and FasL in HL-60 cells through p38 MAPK-mediated c-Jun and ATF-2 phosphorylation. Without specific indication, HL-60 cells were directly treated with 800 nM CTX1 for 4 h, or incubated with 10 μM SB202190 for 1 h and then treated with 800 nM CTX1 for 4 h. (**A**) Effect of CTX1 on Fas and FasL protein expression in HL-60 cells (* *p* < 0.05, CTX1-treated cells compared to untreated control cells). (**B**) Detecting the expression of Fas and FasL mRNAs using qRT-PCR (mean ± SD, * *p* < 0.05). (**C**) CTX1 treatment elicited increase in transcriptional activity of Fas promoter and FasL promoter in HL-60 cells (mean ± SD, * *p* < 0.05). After transfection with indicated promoter constructs for 24 h, the transfected cells were treated with 800 nM CTX1 for 4 h and then harvested for measuring luciferase activity (mean ± SD, * *p* < 0.05). (**D**) Effect of SB202190 on the expression of Fas, FasL, p-c-Jun, and p-ATF-2 in CTX1-treated HL-60 cells (* *p* < 0.05, SB202190/CTX1-treated cells compared to CTX1-treated cells). Depletion of (**E**) c-Jun and (**F**) ATF-2 reduced CTX1-induced Fas and FasL expression. HL-60 cells were transfected with 100 nM control siRNA, c-Jun siRNA, or ATF-2 siRNA, respectively. After 24 h post-transfection, the cells were treated with 800 nM CTX1 for 4 h (* *p* < 0.05, CTX1-treated c-Jun siRNA- or ATF-2 siRNA-transfected cells compared to CTX1-treated control siRNA-transfected cells). (**G**) Effect of SB202190 on the viability of CTX1-treated HL-60 cells (mean ± SD, * *p* < 0.05).

CTX1-treated HL-60 cells showed increased Fas and FasL expression (Figure 7A,B). Compared with untreated cells, CTX1 treatment increased Fas and FasL promoter luciferase activity (Figure 7C). CTX1 increased phosphorylated c-Jun and ATF-2 levels, while SB202190 mitigated this event induced by CTX1 (Figure 7D). Depletion of c-Jun using siRNA abrogated CTX1-induced Fas expression, and CTX1 failed to increase FasL expression in ATF-2 siRNA-transfected cells (Figure 7E,F). Pretreatment with SB202190 protected HL-60 cells from CTX1 cytotoxicity. These results indicate that p38 MAPK-mediated phosphorylation of c-Jun and ATF-2 leads to upregulation of Fas and FasL expression in HL-60 cells after CTX1 treatment. As a result, CTX1 induces the FasL/Fas death pathway in HL-60 cells.

## 4. Discussion

The data in this study demonstrated that CTX1 induced Fas and FasL expression in U937 and HL-60 cells, which thus activates FasL/Fas-mediated death signaling. Mechanistically, CTX1 activated the Ca^2+^/NOX4/ROS/p38 MAPK axis, leading to ATF-2-mediated FasL expression and c-Jun-mediated Fas expression. Because c-Jun, c-Fos, and ATF-2 can form heterodimers or homodimers of AP-1 transcriptional factors [36], we inferred that c-Jun and ATF-2 perform their functions in a homodimer state in CTX1-treated cells. Notably, CTX1 treatment also induced dephoshorylation of ERK and c-Fos. A previous study suggested that c-Fos represses FasL expression in CD4+ T cells [37]. Experimental evidence has demonstrated that c-Fos expression abolishes c-Jun-mediated Fas transcription [38]. Some studies have indicated that arachidonic acid induces the activation of p38 MAPK/ATF-2 and the inhibition of ERK/c-Fos pathways, leading to increased Fas/FasL expression in U937 cells [26]. Other studies have demonstrated that piceatannol-induced ERK and c-Fos inactivation is a prerequisite step in stimulating p38α MAPK/c-Jun- and p38α MAPK/ATF-2-mediated Fas and FasL expression in U937 cells [28]. Therefore, CTX1-induced inactivation of ERK and c-Jun may be critical for the induction of Fas/FasL expression in leukemia cells.

Among the tested *N. atra* CTXs, only CTX1 upregulated Fas/FasL expression. This indicates that the cytotoxicity of CTX isotoxins is not necessarily mediated by the same mechanism. A previous study showed that *N. atra* CTX3 induces autophagy-dependent apoptosis in U937 cells by activating AMPK [7]. Some researchers have reported that *N. mossambica mossambica* CTXs and *N. atra* CTX3 induce mitochondrial fragmentation, leading to the death of neuroblastoma SH-SY5Y cells and H9C2 myoblast cells [11,13]. A prior study indicated that *N. atra* CTX1 induces lysosomal membrane permeability, thereby leading to the death of leukemia K562 and breast cancer MCF-7 cells [12]. Furthermore, the cytotoxicity of *N. haje*, *N. kaouthia*, and *N. oxiana* CTXs in HL-60 cells and lung cancer A549 cells depends on their ability to destroy cell lysosomes [10]. Taken together, these results suggest that CTX-mediated cytotoxicity is dependent on cellular context and cell type.

Experimental evidence has shown that *N. atra* CTX3-induced disruption of the membrane is not exclusively related to its cytotoxicity [7,39]. Compared with native CTX1, SEM-CTX1 showed higher cytotoxicity, while Met-modified CTX1 showed reduced cytotoxicity. Unlike CTX1 and SEM-CTX1, Met-modified CTX1 failed to increase Fas and FasL expression. Previous studies have shown that modification of Met residues reduces the membrane-damaging activity of CTX1 on egg yolk phosphatidylcholine/dimyristoyl phosphatic acid vesicles by approximately 40% [18]. Although *N.* *atra* CTX2, CTX3, CTX4, and CTX5 actively induce the leakage of lipid bilayers [18], they were unable to induce Fas and FasL expression. It appears that CTX1-induced upregulation of these proteins is not related to its membrane-damaging activity. Blocking Asp29 and Asp40 with semicarbazide did not impair CTX1-induced Fas and FasL upregulation, whereas modification of Met24, 26 and 27 abrogated the ability of CTX1 to upregulate Fas/FasL expression. These results indicate that the intact Met residues of CTX1 are crucial for upregulating Fas and FasL expression via the p38 MAPK-mediated pathway. Previous studies have demonstrated that the pore structure formed by CTX3 leads to Ca^2+^ influx in NIH3T3 cells, while CTX2 and CTX4 do not show such an effect [40]. Our data showed that CTX1 induced Ca^2+^ influx in U937 cells. Collectively, these results emphasize that the cytotoxicity of different CTXs can be mediated through different mechanisms.

Importantly, the results of this study do not imply that CTX1 can be used directly to treat leukemia. Previous studies have shown that CTX1 and other *N. atra* CTXs induce hemolysis of RBCs [18]. Other studies have indicated that *N. atra* CTX1 causes necrosis of skeletal muscle cells after intramuscular injection into mice [41]. Therefore, we infer that CTX1 should show cytotoxicity to normal cells when administered systemically. In view of the finding that the intact Met residues at positions 24, 26, and 27 are related to the ability of CTX1 to induce FasL and Fas expression, it is possible to further use the region around the Met residues of CTX1 to develop peptide mimetics to induce Fas-mediated apoptosis in leukemia cells. Since it is feasible for peptidomimetics to improve their specificity and efficacy through structural optimization [42], the optimization of the CTX1 peptidomimetic may retain the ability to induce apoptosis of leukemia cells but avoid the cytotoxic effect of CTX1 on normal cells.

## 5. Conclusions

In summary, our findings demonstrate that *N. atra* CTX1 activates Fas/FasL-mediated U937 and HL-60 cell death. Although *N. atra* CTXs share high degree of sequence conservation, the CTX1-induced death pathway was not observed with other *N. atra* CTXs. These results indicate that slight changes in the fine structure of CTXs greatly affect the mode of their cytotoxic mechanism, and suggest that the diverse activities of CTXs may be due to the activation of different cytotoxic mechanisms in different cells.

## Data Availability

The data that support the findings of this study are available from the corresponding author upon reasonable request.

## Supplementary Materials

The following are available online at https://www.mdpi.com/article/10.3390/cells10082073/s1, Table S1: Primers used for qRT-PCR.
