# Supplementary material for: Naja atra Cardiotoxin 1 Induces the FasL/Fas Death Pathway in Human Leukemia Cells
Source: Cells. 2021 Aug 12;10(8):2073. doi: 10.3390/cells10082073 (PMC8394927; doi:10.3390/cells10082073)
Supplement: Supplementary file 1 [file cells-10-02073-s001.zip › cells-1316056-supplementary.pdf]

Supplementary Table S1. Primers used for qRT-PCR.

Gene	Nucleotide sequence
Fas (forward)	5'-CTCCTACCTCTGGTTCTTACGTCT-3'
Fas (reverse)	5'-GAGCCCCAGCCTTCTCCATG -3'
FasL (forward)	5′- CTCCGAGAGTCTACCAGCCAGATG -3′
FasL (reverse)	5′- GAGTTGGACTTGCCTGTTAAATGGG -3′
GAPDH (forward)	5'-GAAATCCCATCACCATCTTCCAGG-3'
GAPDH (reverse)	5'-GAGCCCCAGCCTTCTCCATG-3'
